# Distress and coping among youth during COVID-19: a national cross sectional study in France

**DOI:** 10.3389/fpsyt.2025.1601358

**Published:** 2025-11-13

**Authors:** Dalila Rezzoug, Isaura Laurent, Mégane Estevez, Bruno Falissard, Thierry Baubet, Enguerrand Habran, Nicolas Oppenchaïm, Stéphanie Vandentorren, Carla De Stefano

**Affiliations:** 1 University Paris 13 Sorbonne Paris Nord, EA 4403 (UTRPP), Bobigny, France; 2 Assistance Publique des Hôpitaux de Paris (AP-HP), Department of Child and Adolescent Psychiatry and General Psychiatry, Avicenne Hospital, Bobigny, France; 3 Centre de Recherche en épidémiologie et santé des populations (CESP), University Paris Saclay, Villejuif, France; 4 Ecole Nationale de Statistique et Analyse de l’Information (ENSAI), Bruz, France; 5 University Bordeaux, Inserm, UMR1219, Bordeaux, France; 6 Centre National de Ressources et de Resilience (CN2R), Lille, France; 7 Fonds Fédération Hospitalière de France (FHF) recherche et innovation, Paris, France; 8 Tours University, UMR CITERES 7324, Tours, France; 9 Santé Publique France, Scientific and International Direction, Saint-Maurice, France; 10 Assistance Publique des Hôpitaux de Paris (AP-HP), Emergency Department (SAMU93), Avicenne Hospital, Bobigny, France

**Keywords:** COVID 19, psychological distress, coping, children, adolescence, mental health

## Abstract

**Background:**

In March 2020, the World Health Organization declared the SARS-CoV-2 outbreak a pandemic. In France, this led to school closures and strict lockdown measures. This unprecedented context disrupted the social lives and mental health of children and liadolescents.

**Methods:**

CONFEADO is a nationwide cross-sectional study designed to assess psychological distress in relation to coping strategies and emotional factors. Conducted between June 9 and September 14, 2020, the study included children aged 9 to 18 and their parents, including youth in child welfare services, all of whom provided informed consent. Data were collected via a self-administered, *ad hoc* web-based questionnaire distributed through national institutions, associations, and social media. For children in welfare services, a paper version was completed with the assistance of childcare professionals. The primary outcome was psychological distress, measured using the Children and Adolescents Psychological Distress Scale-10 (CAPDS-10). Additional variables included coping strategies (behavioral, cognitive, emotional, relational, spiritual), socio-environmental factors (e.g., living in housing with fewer than three rooms, low parental perceived social support), family conflict (e.g., increased conflict or violence), quality of family relationships (e.g., getting along with parents or siblings less than usual), eating habits (e.g., changes in appetite), and school-related stress (e.g., feeling overwhelmed by schoolwork).

**Results:**

A total of 3,148 children and adolescents participated. In bivariate analyses, children who viewed religion as a positive or novel aspect of lockdown (spiritual coping) had a higher likelihood of experiencing moderate distress [OR = 1.26; 95% CI: 0.99–1.60; p = 0.06] and a significantly higher likelihood of experiencing severe distress [OR = 1.99; 95% CI: 1.44–2.74; p < 0.0001]. Relational coping was protective: lower perceived relational support was associated with increased risk of both moderate distress [OR = 0.79; 95% CI: 0.75–0.83; p < 0.0001] and severe distress [OR = 0.55; 95% CI: 0.49–0.61; p < 0.0001]. In multivariate models, severe distress was significantly associated with living in small housing, low parental perceived support, feeling overwhelmed by schoolwork, and appetite changes. Higher levels of behavioral and emotional coping difficulties and increased family conflict were also associated with greater distress.

**Conclusions:**

Coping strategies are key indicators of psychological distress in youth. Assessing how children and adolescents adapt during crises may provide deeper insights than symptom-based approaches alone. The role of spiritual coping in emotional adjustment warrants further clinical and research attention.

## Background

With the onset of the (COronaVIrus Disease 2019) COVID-19 pandemic, lockdowns were implemented globally as a public health strategy (1, 2). However, their psychological, psychiatric, and social consequences —particularly in the longer term — were not fully anticipated (3). A rapid review by Brooks et al. (4) highlighted the significant psychological impact of quarantine, identifying key stressors such as prolonged isolation, fear of infection, frustration, financial losses, and boredom. These stressors contributed to symptoms including depression, insomnia, and post-traumatic stress, as later confirmed in systematic reviews focusing on the general population (5) and on specific vulnerable subgroups, including healthcare workers and patients (6, 7). Children and adolescents, though less studied initially, are particularly vulnerable due to the developmental nature of this life stage. Late childhood and adolescence are key periods for building emotional regulation, interpersonal skills, and coping strategies. These processes are closely tied to daily routines and socialization — elements deeply disrupted by school closures and social distancing. Early studies in youth populations during the pandemic have reported increased levels of anxiety and depressive symptoms (8–10; 11, 12), as well as sleep and appetite disorders (13), fears related to illness (14), and behavioral difficulties, particularly among children with neurodevelopmental disorders (15). In children, perceived pandemic-related stress has been shown to undermine basic psychological needs, particularly autonomy, leading to heightened social anxiety. Complementary findings in female medical students indicate that prolonged stress was associated with increased depressive symptoms, menstrual disturbances, and, most notably, sleep problems. Given the critical role of sleep in emotional regulation and neurodevelopment, especially during childhood and adolescence, sleep disturbances emerge as a key marker of psychological distress (16, 17). Child development takes place within a broader systemic environment, shaped by continuous interactions between various systems (e.g., family, school, and social networks). In a context of chronic stress and social isolation—combined with a loss of autonomy due to lockdown measures—these systems can be significantly disrupted. Children are thus required to draw upon their internal resources to cope with these challenges. Depending on the coping strategies they employ, they may experience varying degrees of psychological distress—or, in some cases, remain unaffected (18).

Conversely, adherence to rules, parental guidance, and peer contact were identified as protective factors that helped mitigate emotional distress (8–11). Further investigations during the pandemic confirmed widespread psychological distress among children and adolescents, often characterized by anxiety, depression, and difficulties with emotional regulation (19, 20). Other studies explored the broader impact on quality of life, sleep, substance use, and family dynamics, showing significant disruptions to youths’ emotional, behavioral, and relational functioning (21–23). However, across this growing body of literature, the severity of psychological distress remains poorly characterized, and coping strategies are rarely addressed from the child’s own perspective.

In France, the first national lockdown—from March 17 to May 10, 2020—included the closure of all schools. To date, only two studies have directly surveyed children and adolescents in France. Our study explored their emotional experiences since the lockdown, focusing on their mental health and the strategies they used to protect their psychological well-being. Another study (PIMS2 CoV19) investigated children’s mindset and experiences related to school learning (24).

CONFEADO is the first large-scale, nationwide French study aiming to:

1. Assess psychological distress in children aged 9 to 18 (primary outcome) definied by Child and Adolescent Psychological Distress Scale – 10 (CAPDS-10) (25). Psychological distress encompasses a range of symptoms, such as anxiety, depression, somatic complaints, and manifestations of aggressive behavior.

2. Identify the coping strategies (behavioural, cognitive, emotional, relational, spiritual) they used during the lockdown (secondary outcome).

We hypothesize that coping strategies are associated with levels of psychological distress, independently of environmental influences. We hypothesize that external environmental factors, which require adaptive capacities, are associated with higher levels of psychological distress.

## Methods

### Study design

CONFEADO (CONFinement Enfants et ADOlescents) is a nationwide, cross-sectional study conducted in France, targeting children and adolescents aged 9 to 18 years, as well as their parents or legal guardians. Eligibility criteria included the ability to provide informed assent or consent; youth under the care of the French child welfare services were also included. Children under 9 or over 18 years of age were excluded.

Data collection took place between June 9 and September 14, 2020, immediately following the first national lockdown. Although schools had officially reopened, most children and adolescents had not resumed in-person classes. The recruitment period spanned the final month of the academic year, the summer holidays, and the start of the new school year—a time marked by increased anxiety among adults and adolescents following the government’s announcement of a full return to in-person schooling.

The study was approved by a French Research Ethics Committee (No. 2020-A01342.37). Participants were informed of the study’s objectives and ethical approval. The survey was anonymous, and no incentives were offered.

### Data collection and outcomes

#### Recruitment procedure

The link to the questionnaire was sent by national institutions or associations and via social media (e.g. Twitter, Facebook). For children in welfare services, the questionnaire was available on paper through childcare workers.

### Quantitative and qualitative assessment tools

#### General questionnaire

To construct the questionnaire, we used sociodemographic items validated in the literature (26) alongside questions specifically adapted to the lockdown context for assessing emotional state and coping strategies. The questionnaire included psychometric scales and items on mental health. The children and adolescents responded via an online or paper questionnaire. The questionnaire was made anonymous, standardized, and developed from a multidisciplinary perspective (public health, epidemiology, psychology, psychiatry and sociology). It had a section for adult caregivers, followed by a section for youth. A system of vocal synthesis was provided in case of illiteracy. The questionnaire completed by parents collected socio-demographic data (gender, age, municipality of residence, employment status, occupation, diploma, nationality, perception of the financial status of the household) and data on the living conditions during the lockdown period and the impact of the pandemic within the household (type and size of housing, financial impact, people who were ill with, or died from, Covid-19 in the family circle).

The questionnaire completed by children collected:

- socio-demographic data (gender, age, family bilingualism, etc.)
- living conditions (housing, neighborhood)
- activities during lockdown (time spent on schoolwork, screen time)
- relationships during lockdown (contact with friends, cyberbullying, relationships within the family)
- data related to Covid-19 (close family/relative infected or hospitalized)
- coping strategies during lockdown (positive/negative points of lockdown, independence)
- child’s/adolescent’s general physical condition and emotional state (sleep, appetite, emotions upon waking-up and at bedtime).

### Study outcomes

#### Primary outcome

The Children and Adolescent Psychological Distress Scale (25) (CAPDS-10) was designed and validated previously for the needs of this research (primary outcome). We targeted ten items reporting on aspects of psychological distress: anxiety, stress, emotional regulation, depressive symptoms, symptoms expressed through body language and the impact of aggressiveness on relationships. It is a self-report tool with 10 items to which the child can answer “Never”, “Sometimes”, “More than half of the time” or “Almost every day”. This leads to a score between 0 and 3 for each answer, and a total score between 0 and 30. Children are instructed to answer based on how they have felt over the past two weeks. The higher the score, the more intense the psychological distress.

##### Exploratory outcomes

We took into consideration the following variables when analyzing the primary outcome:

- The adult caregiver’s socio-demographic characteristics, data on the living conditions during lockdown and its impact via the parent/adult caregiver questionnaire
- The child’s/adolescent’s socio-demographic characteristics and data on the living conditions and its impact during lockdown via the child-adolescent questionnaire

##### Secondary outcome

For this objective, we created five coping scores and one family conflict score and we used items from the questionnaire and the Child and Youth Resilience Measure (CYRM-R) (27):

- Emotional coping (“feeling things without saying them”, “keeping secrets inside”, reporting “stress”, “loneliness”, and “boredom” as negative points related to the lockdown. CYRM-R: “I talk to my family about how I feel” and “I feel safe when I am with my family”). We coded positively the fact of keeping things to oneself and feeling negative emotions. In the CYRM-R scale, communicating emotions and feeling safe were rated positively on a score of 1 to 5. After reversing this coding and reducing the score to between 0 and 1, each variable had the same weight in the construction of the emotional coping score and went in the same direction. Thus, the higher the emotional coping score, the more children keep what they feel to themselves and the more the children experience negative emotions. For this coping strategy (emotional coping), the scoring is reversed compared to other strategies: a higher score indicates less effective coping.

- Behavioral coping (doing things on your own, cooking, gardening, doing crafts, playing, reading, etc.). This coping score also contained the variable “Since you have to stay at home, do you eat too much?” Eating more was considered as an activity performed to manage stress). The coding was 0 and 1.
- Relational coping (“I am in touch with my friends”, “I play with my siblings or other children”, “I spend time with my family”. CYRM-R: “I feel supported by my friends”, “My family/friends will always be ready to support me in difficult times”). The coding was reduced to between 0 and 1. Thus, the higher the relational coping score, the more the children benefit from the social relationships around them);
- Cognitive coping (“I don’t see any negative points [to the lockdown]”, “I see no positive points”, “I’m learning from my parents with the lockdown”, “I’m discovering new interests with the lockdown”, “The lockdown hasn’t brought me any novelty”, and finally “Since I am at home, I’d rather be alone than be with others”). The coding was done in coherence with a cognitive bias called “negativity bias” (28, 29; 30), which entails giving more importance to negative experiences and remembering them more. Children who said that they did not see any positive points or novelty from the lockdown and preferred to be left alone will get extra points;
- Spiritual coping is centered around two variables: “Are religious practices a positive/new feature of the lockdown?” If the child answers “No” to these two questions, the score is 0. If the child answers “Yes” to either question, the score is 1, and if religion is both a novelty and a positive feature of the lockdown for the child, the score is 2.
- For the family conflict score, four variables were used: “A negative point of the lockdown was that there was more conflict between adults/between brothers, sisters, children/between parents and children/more violence in the family”. The family conflict score is between 0 and 4.

We hypothesized that children who engaged in few activities, perceived low social support, were unable to identify positive aspects of the situation, felt they were not learning from their parents, and did not share their emotional experiences were at greater risk of experiencing moderate to severe psychological distress. Conversely, children who reported discovering religion or spirituality appeared to be at lower risk of such distress.

Trait anxiety was assessed in children. We used the State-Trait Anxiety Inventory for Children (STAIC) (31), a self-administered questionnaire consisting of 10 items rated on a 3-point Likert scale. The total score ranges from 20 to 60, with higher scores reflecting greater levels of trait anxiety.

### Statistical analysis

We conducted a logistic regression in bivariate mode in order to validate the link between the psychological distress and coping variables. This allowed us to select the features that influence the target variables according to the Odds Ratio (OR). Then, we checked the confounding factors with a multivariate approach.

We were able to handle the missing data using multiple imputation with chained equations.

As part of this multiple imputation, m complete datasets (m=30) were created by simple imputation, and the results of the models run on these m datasets were then compiled according to Rubin’s law. Specifically, pooled estimates were obtained by averaging point estimates across datasets, while standard errors were adjusted to incorporate both within- and between-imputation variance, thereby ensuring valid statistical inference under the assumption of missing at random (MAR).

After validating the multiple imputation procedure, we performed a stepwise variable selection process to build the final model. The stepwise method iteratively adds or removes variables based on predefined statistical criteria (e.g., Akaike Information Criterion [AIC] or p-values), and can proceed in a forward, backward, or bidirectional manner. This procedure facilitates the development of models that balance explanatory power with model simplicity. The variables considered in this procedure included: age, sex, date of questionnaire completion, parental education level, housing overcrowding, family structure, housing type, number of rooms, access to a computer, internet connection, number of siblings, household income, income loss due to the pandemic, nationality, other languages spoken at home, perceived social support, hospitalization or death of a close relative, food insecurity, significant events reported by parents and children, child’s medical or psychiatric history, time spent outdoors, use of social media, gaming console usage, screen time (TV/videos/movies), frequency of information seeking, frequency of recreational activities, potential social isolation, academic support, feelings about schoolwork, cyberbullying, sleep duration, sleep disturbances, appetite, relationship with siblings, relationship with parents, doing by yourself, emotional coping score, cognitive coping score, behavioral coping score, relational coping score, spiritual coping score, family conflict score, resilience score, and trait anxiety score.

Following this stepwise selection, variables with a significance level below 20% in univariate regression were retained in the multivariable model: sex, number of rooms, income loss due to the pandemic, social support, significant events reported by parents and children, child’s medical history, feelings about schoolwork, appetite, relationship with siblings, relationship with parents, doing by yourself, emotional coping score, behavioral coping score, family conflict score, and trait anxiety score. Cyberbullying, age, and hospitalization or death of a close relative were included as forced variables.

The final model was a multinomial logistic regression (polytomous model) examining psychological distress categorized into three levels: no to mild distress (score 0–9), moderate distress (score 10–18), and severe distress (score ≥19). The analysis was restricted to respondents who completed the CAPDS-10 scale (N = 3148). All analyses were conducted using R version 4.3.0, with a significance threshold set at α = 0.05.

## Results

### Sample characteristics and population selection

A total of 5,327 participants provided consent (25). Of these, 3,148 participants were included in the analysis. Exclusions were due to age criteria (1,357 participants were under 9 or over 18 years old), incomplete questionnaires (42 participants), or extreme outliers and missing data in the initial section (30 participants).

The initial analyses of the CONFEADO study described the socio-demographic characteristics of the sample (25). Our population consisted of 922 children—426 (13%) aged 9–11 years and 496 (16%) aged 12–14 years—and 2,226 adolescents (71%) aged 15–18 years. More girls (N = 2,206; 70%) than boys (N = 942; 30%) responded to the survey.

Among the study population, 81 participants (2%) were under the care of child welfare services: 23 (28%) aged 9–11 years, 26 (32%) aged 12–14 years, and 32 (40%) aged 15–18 years. Again, more girls (N = 50; 62%) than boys (N = 31; 38%) responded. Among these, 40% (N = 32) were living in group homes, 50% (N = 40) in foster families, and 10% (N = 8) in non-profit housing settings.

### Distress based on socio-environmental and psychological variables

In our sample, 61% of children and adolescents (N = 1929) reported no to mild psychological distress, 31% reported moderate distress (N = 979), and 8% reported severe distress (N = 240).

Regarding living conditions, the risk of severe psychological distress was significantly higher among girls (p<0.0001), children and adolescents living in overcrowded housing (p<0.0001), those with poor or no internet access (p<0.0001), those with two foreign-born parents (p<0.0001), those speaking a language other than French at home (p<0.0001), those who did not perceive social support as available (p<0.0001), and those experiencing food insecurity (p<0.0001).

In terms of psychological and individual factors, severe distress was more prevalent among children and adolescents who felt overwhelmed by schoolwork (p<0.0001), had a relative infected or hospitalized due to COVID-19 (p<0.0001), were victims of cyberbullying (p<0.0001), had higher trait anxiety scores (p<0.0001), whose parents reported a stressful and/or painful event for themselves or their child (p<0.0001), had pre-existing emotional or developmental disorders impacting daily life prior to the lockdown (p<0.0001), slept less than 8 hours and 30 minutes per night (p<0.0001), had higher disturbed sleep scores (p<0.0001), reported reduced or no appetite (p<0.0001), reported poorer relationships with parents or siblings (p<0.0001), had a high family conflict score (score of 4) (p<0.0001), or felt less autonomous than usual (p<0.0001).

### Distress based on coping strategies

**Table 1** presents the distribution of coping strategies and the family conflict score. Regarding spiritual coping, the majority of participants (93%, *n* = 2,926) had a score of 0, while 6% (*n* = 181) scored 1, and 1% (*n* = 41) scored 2.

**Table 1:** Coping variables and the family conflicts score distribution.

Type of coping	Min	Median	Mean	Max
Emotional Coping	0.00	2.50	2.75	7.00
Cognitive Coping	-3.00	0.00	0.34	3.00
Comportemental Coping	0.00	5.00	5.58	16.00
Relational Coping	0.00	3.00	3.10	7.00
Family conflicts score	0.00	0.00	0.55	4

**Figure 1:**
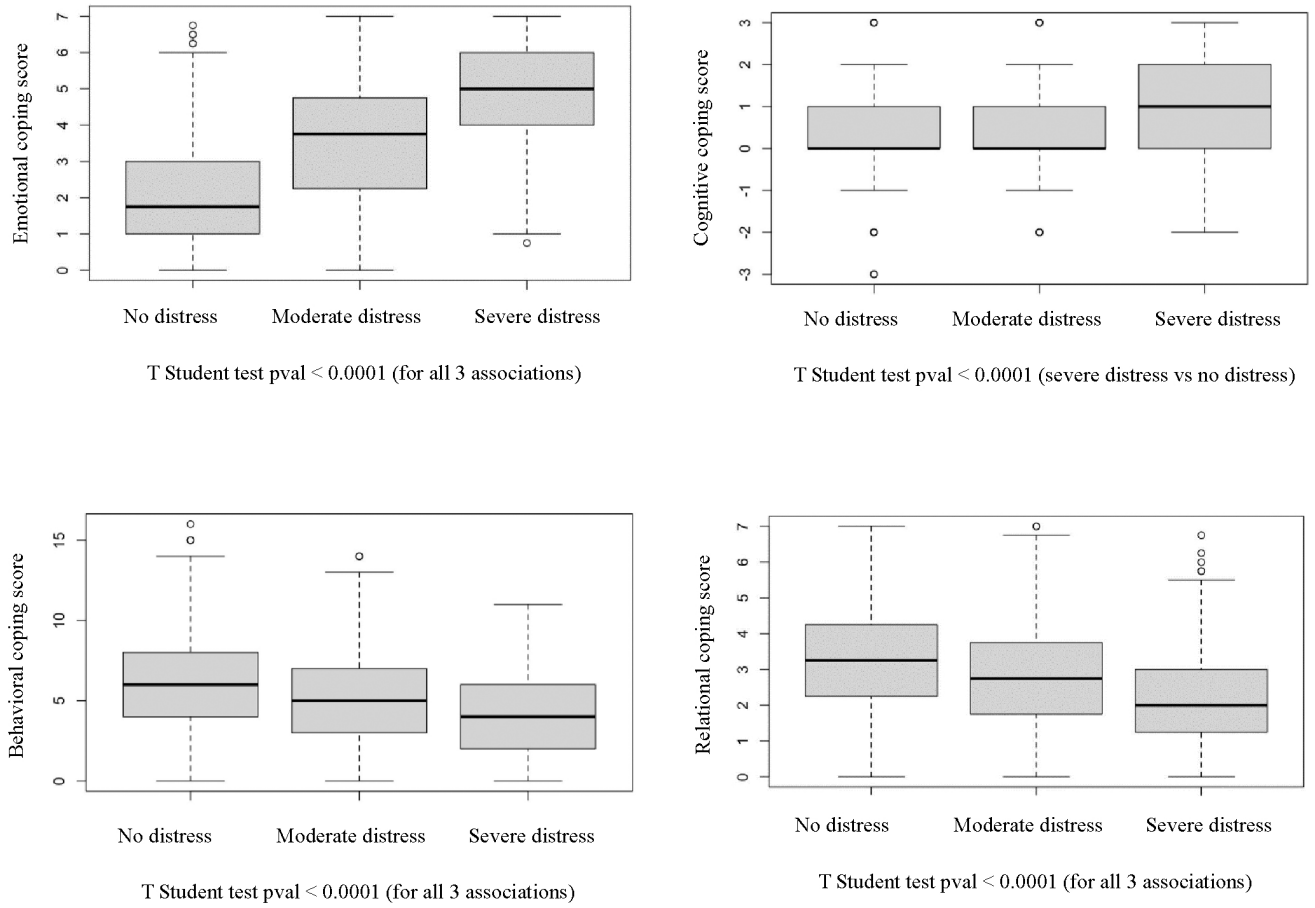
Bivariate analysis of psychological distress and coping variables.

**Figure 1** illustrates the bivariate associations between psychological distress and coping strategies.

In the multinomial regression analysis, associations between coping strategies and levels of psychological distress were assessed. For emotional coping, children who tended to keep their emotions to themselves were more likely to present with moderate [OR = 2.02, 95% CI: 1.90–2.14, *p* < 0.0001] or severe distress [OR = 3.40, 95% CI: 3.04–3.81, *p* < 0.0001]. Notably, the emotional coping score was inversely coded, meaning that a higher score indicated less adaptive coping.

Regarding cognitive coping, children who failed to identify any positive aspects or novelty in the lockdown period had an increased likelihood of moderate [OR = 1.22, 95% CI: 1.14–1.31, *p* < 0.0001] or severe distress [OR = 1.90, 95% CI: 1.67–2.17, *p* < 0.0001].

For behavioral coping, fewer engagement in activities such as cooking, playing, or reading was associated with greater odds of moderate [OR = 0.90, 95% CI: 0.87–0.92, *p* < 0.0001] and severe distress [OR = 0.76, 95% CI: 0.71–0.80, *p* < 0.0001].

Regarding relational coping, children who perceived less support from their environment were more likely to experience moderate [OR = 0.79, 95% CI: 0.75–0.83, *p* < 0.0001] or severe distress [OR = 0.55, 95% CI: 0.49–0.61, *p* < 0.0001].

Finally, for spiritual coping, children who perceived religion or spirituality as a new or positive element during lockdown showed a trend toward moderate distress [OR = 1.26, 95% CI: 0.99–1.60, *p* = 0.06] and a significantly higher likelihood of severe distress [OR = 1.99, 95% CI: 1.44–2.74, *p* < 0.0001].

**Table 2** displays the results of the regression analysis for all coping strategies.

**Table 2:** Regression analysis of psychologic distress and coping strategies.

Distress Level	Coping Strategy	OR [95% CI]	p-value
Moderate distress	Emotional coping score	1.98 [1.86 – 2.10]	< 0.0001
	Behavioral coping score	1.01 [0.92 – 1.11]	0.82
	Cognitive coping score	0.95 [0.91 – 0.98]	0.004
	Relational coping score	0.92 [0.86 – 0.98]	0.014
	Spiritual coping score	1.19 [0.90 – 1.56]	0.027
	Emotional coping score	3.11 [2.77 – 3.49]	< 0.0001
Severe distress	Behavioral coping score	1.28 [1.08 – 1.52]	0.004
	Cognitive coping score	0.92 [0.85 – 0.99]	0.020
	Relational coping score	0.76 [0.67 – 0.87]	< 0.0001
	Spiritual coping score	2.07 [1.40 – 3.06]	< 0.0001

### Psychological distress model

After stepwise selection, the final model comprised two components: the first estimated parameters associated with the risk of moderate distress versus low distress, and the second focused on severe distress versus low distress, with low distress as the reference category.

The main differences were that the variables “number of rooms” and “social support” were significant only for severe distress, while behavioral coping was significant at the 10% level only for moderate distress but reached the 5% significance threshold for severe distress. Moreover, odds ratios for variables significant at both distress levels were higher for severe distress, indicating stronger effects.

Risk factors for severe distress included:

- Living in a small dwelling (two rooms or fewer)
- Lack of social support perceived by parents (feeling isolated and unsupported)
- Worse-than-usual relationships with parents
- Worse-than-usual relationships with siblings
- Experiencing family conflicts
- Disturbed appetite [OR = 2.38, 95% CI: 1.51–3.75, p < 0.001]
- Feeling completely or sometimes overwhelmed by homework [OR = 3.10, 95% CI: 1.66–5.77, p < 0.001]
- Doing by yourself less than usual
- History of psychological or developmental disorders [OR = 2.03, 95% CI: 1.32–3.13, p = 0.001]
- High trait anxiety score [OR = 1.31, 95% CI: 1.27–1.36, p < 0.001]
- High emotional coping score
- Low behavioral coping score

Sex and age, significant in univariate analyses, were no longer significant in the full multivariate model. However, their effects may be mediated by other variables: older children are more likely to have a history of psychological disorders, and adolescents tend to feel more overwhelmed by homework than younger children. Age is also associated with parent-child relationship quality and appetite disturbances: adolescents report worse relationships with parents compared to younger children (p = 0.0007, chi-square test), and more frequently report reduced appetite (p < 0.0001, chi-square test). Moreover, adolescents report feeling completely overwhelmed by homework more often than children (p = 0.0003) and have a higher prevalence of prior psychological disorders (p < 0.0001). These associations may explain why age itself was not significant in the final model.

The emotional coping score was highly significant for both moderate and severe distress. Specifically, children who tend to keep their feelings to themselves and communicate little about their emotions had increased odds of moderate distress [OR = 1.35, 95% CI: 1.25–1.45, p < 0.0001] and severe distress [OR = 1.64, 95% CI: 1.43–1.89, p < 0.001]. Behavioral coping was significant only for severe distress: children engaging in fewer activities at home (e.g., cooking, playing, reading) were more likely to experience severe distress [OR = 1.89, 95% CI: 1.83–1.96, p = 0.002].

Relational coping was not retained in the multivariate model despite significance in univariate analyses. This is likely because related constructs—such as relying on family support—are captured by other variables in the model, including relationships with parents and siblings, family conflict scores, and emotional coping, which assesses communication with close ones.

Overall, family relationships play a major role in explaining psychological distress: the three family-related variables—”Getting along with parents” (moderate distress OR = 1.88, 95% CI:1.42–2.49, p < 0.0001; severe distress OR = 2.72, 95% CI:1.74–4.25, p < 0.001), “Getting along with siblings” (moderate distress OR = 1.50, 95% CI: 1.12–2.01, p = 0.006; severe distress OR = 2.08, 95% CI:1.29–3.35, p = 0.002), and family conflict score (moderate distress OR = 1.38, 95% CI:1.21–1.57, p < 0.001; severe distress OR = 1.76, 95% CI: 1.43–2.15, p < 0.001)—were all highly significant. Thus, family relationships, both with parents and siblings, have a strong impact on mental health.

Sibling relationships can impact a child’s psychological well-being as much as relationships with parents. These effects may have been intensified by the lockdown, which forced children to live continuously with siblings without interruption.

Cognitive coping, although significant in univariate analyses and in a regression including only the five coping strategies, was not significant in the full multivariate model. This is likely because aspects covered by this strategy—for example, learning from parents or preferring solitude—are captured by other variables such as parent-child relationship quality or “doing things by yourself” (severe distress OR = 2.03, 95% CI: 1.12–3.68, p = 0.019).

Regarding spiritual coping, the proportion of children reporting religion as a new and/or positive practice since the lockdown was very low, which may explain why this coping strategy was not retained in the final model. Nevertheless, it was significant in univariate analyses, with a significantly higher proportion of children with moderate to severe distress among those who reported religion as a novelty and/or positive aspect of the lockdown.

**Table 3** presents the multivariate analysis of moderate psychological distress.

**Table 3:** Multivariate analysis of moderate psychological distress.

Variable	Category	OR [CI 95%]	P value	
(Intercept)		0.00 [0.00 – 0.00]	0,000	
Sex	Boy	0.81 [0.63 – 1.04]	0.093	*
Age	12–14 years	0.86 [0.58 – 1.27]	0.452	
Age	15–18 years	0.96 [0.70 – 1.33]	0.828	
Affected incomes	Yes decreased	0.83 [0.62 – 1.13]	0.233	
Number of rooms	3 or more	0.83 [0.52 – 1.35]	0.459	
Social Support	Yes	0.91 [0.66 – 1.24]	0.545	
Loved one hospitalized or deceased	Yes	0.93 [0.73 – 1.19]	0.583	
Cyberbullying	Yes	1.27 [0.71 – 2.29]	0.418	
Get along with parent	Less than usual	1.88 [1.42 – 2.49]	0.000	***
Get along with siblings	No brother or sister	0.81 [0.60 – 1.09]	0.161	
	Less than usual	1.50 [1.12 – 2.01]	0.006	***
Family conflict score		1.38 [1.21 – 1.57]	0.000	***
Appetite	No or less than usual	1.71 [1.34 – 2.19]	0.000	***
	Yes more than usual	1.48 [1.13 – 1.95]	0.005	***
Feeling about homework	Completely overwhelmed	2.21 [1.48 – 3.31]	0.000	***
	Sometimes overwhelmed	1.49 [1.17 – 1.91]	0.001	***
	Very comfortable	0.98 [0.70 – 1.37]	0.910	
Doing things by yourself	Less than usual	1.41 [0.95 – 2.11]	0.091	*
Particular event according to the child	Yes	1.41 [1.14 – 1.75]	0.002	***
History of disorder	Yes	1.61 [1.25 – 2.09]	0.000	***
STAIC score		1.13 [1.11 – 1.15]	0.000	***
Emotional coping score		1.35 [1.25 – 1.45]	0.000	***
Behavioral coping score		0.97 [0.93 – 1.00]	0.086	*

* significant p<0.05. *** significant p<0.001.

**Table 4** presents the multivariate analysis of severe psychological distress.

**Table 4:** Multivariate analysis of severe psychological distress.

Variable	Category	OR [95% CI]	P-Value	
Sex	Boy	1.29 [0.77 – 2.15]	0.328	
Age	12–14 years	1.49 [0.61 – 3.66]	0.385	
Age	15–18 years	0.68 [0.31 – 1.51]	0.345	
Affected incomes	Yes decreased	1.49 [0.90 – 2.45]	0.121	
Number of rooms	3 or more	0.42 [0.20 – 0.90]	0.026	*
Social Support	Yes	0.56 [0.35 – 0.89]	0.013	*
Loved one hospitalized or deceased	Yes	0.88 [0.56 – 1.38]	0.578	
Cyberbullying	Yes	1.20 [0.55 – 2.65]	0.644	
Get along with parent	Less than usual	2.72 [1.74 – 4.25]	0.000	***
Get along with siblings	No brother or sister	0.85 [0.47 – 1.54]	0.596	
	Less than usual	2.08 [1.29 – 3.35]	0.002	***
Family conflict score		1.76 [1.43 – 2.15]	0.000	***
Appetite	No or less than usual	2.38 [1.51 – 3.75]	0.000	***
	Yes more than usual	1.96 [1.19 – 3.22]	0.008	***
Feeling about homework	Completely overwhelmed	3.10 [1.66 – 5.77]	0.000	***
	Sometimes overwhelmed	1.69 [1.02 – 2.78]	0.039	*
	Very comfortable	1.85 [0.90 – 3.81]	0.095	.
Doing things by yourself	Less than usual	2.03 [1.12 – 3.68]	0.019	*
Particular event according to the child	Yes	1.60 [1.01 – 2.51]	0.043	*
History of disorder	Yes	2.03 [1.32 – 3.13]	0.001	***
STAIC score		1.31 [1.27 – 1.36]	0.000	***
Emotional coping score		1.64 [1.43 – 1.89]	0.000	***
Behavioral coping score		0.89 [0.83 – 0.96]	0,002	***

* significant p<0.05. *** significant p<0.001.

## Discussion

This study demonstrated that severe psychological distress in children and adolescents was associated with socio-environmental factors; pre-existing emotional, affective, or developmental disorders; emotional and behavioral coping strategies; a deterioration in family and social relationships; feelings of being overwhelmed by academic demands; and disturbances in appetite. These findings are in line with previous international studies conducted in various countries during the COVID-19 pandemic (19–23, 32–35).

For example, a European longitudinal study focusing on Italy, Spain, and Portugal highlighted the prolonged nature of the COVID-19 crisis and its heterogeneous impacts over time and across regions, depending on the stringency of public health restrictions. Anxiety and depressive symptoms, sleep disturbances, eating behavior disorders, and cognitive symptoms (such as difficulties with memory, concentration, and attention) were found to decrease over time, yet remained elevated six months after the onset of the pandemic—corresponding to the end of our recruitment period (36). These findings reflect the persistence of psychological symptoms beyond the peak of restrictive measures, in line with our own results.

Furthermore, living conditions and parents’ socio-demographic characteristics have an impact on children’s health behaviors and psychological needs (26, 37–39). In addition, Mbithi et al. (40) identified key factors associated with poor mental health in children and adolescents, including loneliness, school closures, COVID-19-related stressors, and family conflicts. These findings are consistent with our own results (40).

Girls, children and adolescents from single-parent families, as well as those with mentally ill or highly burdened parents, were found to be particularly at risk (41). Furthermore, regarding parental burnout, Martinez-Seda et al. demonstrated that adverse experiences reported by parents in relation to the pandemic had both direct and indirect effects on children’s mental health. These effects were mediated by parental stress and psychopathology (42). In our study, low perceived social support among parents was associated with severe psychological distress in children and adolescents.

Some previous studies have looked at children’s coping strategies during the pandemic (13, 43–49). Disruptions to daily life led to feelings of boredom and purposelessness. Limitations in social interaction led to loneliness and an increase in screen time, particularly when seeking social ties with peers (44). Although a stressful and unexpected event can itself lead to negative consequences, the way in which we adapt to it from a cognitive, behavioral and emotional point of view modulates the impact of these consequences in the sense of worsening or improving (50). Indeed, strategies of avoidance, denial, internalization, substance use and overexposure to screens have a harmful impact on mental health, whereas strategies of problem-solving, acceptance, action and cognitive restructuring are more correlated with better psychological states (43, 48). Despite the uncontrollable nature of pandemic-related stressors, if we look at coping strategies from the point of view of engagement or disengagement, or from the active or non-active point of view, it is the activity and engagement strategies that are most relevant, from the behavioral, cognitive and emotional points of view (51). Indeed, our findings show that engaging in activities serves as a protective factor.

On the other hand, in natural disaster situations (e.g. earthquakes), certain active behavioral strategies put adolescents at risk of psychological distress, as they expose them to traumatic risk when they take on a rescue role (50). Our study confirms that when children do little, do not share their emotions and feelings with those around them, do not get along as well as usual with their parents and siblings, and have less autonomy than usual, they are at greater risk of severe distress.

This result opens up the prospect of monitoring parents who are considered less informative for internalized disorders (52, 53). Studies analyzing parent-child agreement on the presence of psychopathology (anxiety-depressive syndrome, suicidal thoughts, Post Traumatic Stress Disorder (PTSD), Generalized Anxiety Disorder (GAD), Attention Deficit and Hyperactivity Disorder (ADHD), behavioral disorders) report weak to moderate levels of agreement (54–56).

Being able to observe that children are keeping things to themselves, enables those around them to spot an internalized factor associated with psychological distress. Furthermore, in our bivariate analysis, we found that when children saw religion as a novelty and a positive point of lockdown, there was a risk of being more severely distressed.

In our bivariate analysis, a statistically significant association was found between spiritual coping, moderate distress and severe distress, with a higher OR in severe distress. For children and adolescents in distress, spiritual coping is a new and beneficial element. Other authors who have studied children’s spiritual coping with stressful situations (school bullying, natural disasters, pandemics) highlight the effectiveness of religious practice as a coping strategy, but do not question the novelty of turning to religion (49, 50, 57).

Even if, in our multivariate model, spiritual coping is not statistically associated, this variable remained an element that we feel is relevant to the identification of psychological distress in children and adolescents. In our sample, the number of participants who reported religion as a positive aspect or a new personal resource was limited. Nevertheless, this variable appears clinically relevant, as it introduces a novel and somewhat counterintuitive dimension. This observation warrants further investigation in larger studies. By contrast, in the field of psychopathology, a body of scientific work has examined the onset of psychosis and experiential self-disturbances, suggesting that sudden religious conversion may constitute a manifestation of self-disturbance and a potential risk factor for mental health during developmental age, particularly in adolescence (58, 59).

Limitations must be reported. Recruitment via web questionnaire may constitute a bias related to education level, literacy and access to digital tools. The main one is that the sample is not representative. In addition, we observed a more pronounced over-representation of girls in the adolescents surveyed. Nevertheless, that breakdown is present in all major epidemiological studies (50, 51).

Regarding the recruitment period, we acknowledge that the timing (June to September 2020) may have influenced the content of the responses provided. Recruitment began shortly after the end of the lockdown and extended over a two-month period marked by significant contextual shifts, including the end of the school year, summer holidays, and the beginning of the new academic year. Therefore, the specific timing at which participants completed the questionnaire may have affected their responses.

Moreover, we did not use a standardized scale to assess stress and its impact in children and adolescents; some items, such as those addressing coping strategies, were developed *ad hoc* specifically for this study.

Our study has several strengths. It is a nationwide French study assessing mental health and experiences following the lockdown, based on self-reports from children and adolescents. This approach provided valuable insights into the children’s perspectives regarding their living conditions and emotional experiences.

Regarding our objectives, we asked children and adolescents to refer specifically to the beginning of the lockdown, which allowed them to anchor their responses in relation to this time point. Several questions were explicitly linked to this period, such as: “Since the lockdown, have you eaten more, the same, or less than before?” This suggests that participants used this temporal reference to guide their answers, indicating a connection between their perceptions and the COVID-19 lockdown as well as the broader pandemic experience, which was the dominant context during this time.

Qualitative questions were also posed, and their analysis will be the subject of a separate publication, further enriching the understanding of children’s and adolescents’ experiences. From a theoretical perspective, in such an unprecedented, distressing, and intense situation at both collective and individual levels, experience becomes saturated by the new circumstances. Accordingly, we consider that our results are closely linked to the consequences of the pandemic.

This study moves beyond a focus on symptoms and psychopathological diagnoses in children to give them a voice and understand how they react and adapt to such situations. This approach opens up a clinical perspective aimed at identifying vulnerable profiles characterized by ineffective coping strategies. Such a clinical framework is particularly relevant in contexts of collective crises that impose prolonged social disruption (e.g., health emergencies, natural disasters, terrorism, armed conflicts) and/or family events that disturb family dynamics due to the shock they cause.

## Conclusions

Our results identified emotional coping strategies that may increase vulnerability in children and adolescents, as well as behavioral coping mechanisms that help safeguard their mental health since the onset of the COVID-19 pandemic.

Recognizing these factors can inform targeted clinical and public health interventions in similar situations by providing adults with guidance on supportive behaviors and by identifying adolescent isolation as a key risk factor. Regarding family relationships, enhancing access to and awareness of national prevention platforms for children and adolescents experiencing intrafamilial violence is crucial. Looking ahead, teachers could be trained and sensitized to effective behavioral coping strategies and equipped with tools to support children’s emotional regulation, given their daily contact with these populations.

From a research perspective, further studies are warranted on coping strategies, with particular emphasis on spiritual coping.

## Data Availability

The data analyzed in this study is subject to the following licenses/restrictions: The data that support the findings of this study are available on request from the corresponding author. The data are not publicly available due to privacy or ethical restrictions. Requests to access these datasets should be directed to carla.destefano85@gmail.com.

## References

[1] Barbera J Macintyre A Gostin L Inglesby T O'Toole T DeAtley C . Large-scale quarantine following biological terrorism in the United States: scientific examination, logistic and legal limits, and possible consequences. JAMA. (2001) 286:2711−17. doi: 10.1001/jama.286.21.2711, PMID: 11730447

[2] CDC . Isolation. In: Centers for Disease Control and Prevention (2023). Available online at: https://www.cdc.gov/coronavirus/2019-ncov/your-health/isolation.html. et CDC.

[3] Hotopf M Bullmore E O’Connor RC Holmes EA . The scope of mental health research during the COVID-19 pandemic and its aftermath. Br J Psychiatry. (2020) 217:540−42. doi: 10.1192/bjp.2020.125, PMID: 32493516 PMC7330278

[4] Brooks SK Webster RK Smith LE Woodland L Wessely S Greenberg N . The psychological impact of quarantine and how to reduce it: rapid review of the evidence. Lancet. (2020) 395:912−20. doi: 10.1016/S0140-6736(20)30460-8, PMID: 32112714 PMC7158942

[5] Xiong J Lipsitz O Nasri F Lui LMW Gill H Phan L . Impact of COVID-19 pandemic on mental health in the general population: A systematic review. J Affect Disord. (2020) 277:55−64. doi: 10.1016/j.jad.2020.08.001, PMID: 32799105 PMC7413844

[6] Krishnamoorthy Y Nagarajan R Saya GK Menon V . Prevalence of Psychological Morbidities among General Population, Healthcare Workers and COVID-19 Patients amidst the COVID-19 Pandemic: A Systematic Review and Meta-Analysis. Psychiatry Res. (2020) 293:113382. doi: 10.1016/j.psychres.2020.113382, PMID: 32829073 PMC7417292

[7] Wathelet M Duhem S Vaiva G Baubet T Habran E Veerapa E . Factors associated with mental health disorders among university students in France confined during the COVID-19 pandemic. JAMA Netw Open. (2020) 3:e2025591. doi: 10.1001/jamanetworkopen.2020.25591, PMID: 33095252 PMC7584927

[8] Oosterhoff B Palmer CA Wilson J Shook N . Adolescents’ Motivations to engage in social distancing during the COVID-19 pandemic: associations with mental and social health. J Adolesc Health. (2020) 67:179−85. doi: 10.1016/j.jadohealth.2020.05.004, PMID: 32487491 PMC7205689

[9] Zhou S-J Zhang L-G Wang L-L Guo Z-C Wang J-Q Chen J-C . Prevalence and socio-demographic correlates of psychological health problems in Chinese adolescents during the outbreak of COVID-19. Eur Child Adolesc Psychiatry. (2020) 29:749−58. doi: 10.1007/s00787-020-01541-4, PMID: 32363492 PMC7196181

[10] Seçer İ Ulaş S . An investigation of the effect of COVID-19 on OCD in youth in the context of emotional reactivity, experiential avoidance, depression and anxiety. Int J Ment Health Addict. (2021) 19:2306−19. doi: 10.1007/s11469-020-00322-z, PMID: 32837429 PMC7293436

[11] Xie X Xue Q Zhou Y Zhu K Liu Q Zhang J . Mental health status among children in home confinement during the coronavirus disease 2019 outbreak in Hubei Province, China. JAMA Pediatr. (2020) 174:898−900. doi: 10.1001/jamapediatrics.2020.1619, PMID: 32329784 PMC7182958

[12] Liu X Luo W-T Li Y Li J Ye L Liu D . Psychological status and behavior changes of the public during the COVID-19 epidemic in China. Infect Dis Poverty. (2020) 9:58. doi: 10.1186/s40249-020-00678-3, PMID: 32471513 PMC7256340

[13] Jiao W-Y Wang L-N Liu J Fang S-F Jiao F-Y Pettoello-Mantovani M . Behavioral and emotional disorders in children during the COVID-19 epidemic. J Pediatr. (2020) 221:264–266.e1. doi: 10.1016/j.jpeds.2020.03.013, PMID: 32248989 PMC7127630

[14] Casanova M Bagliacca EP Silva M Belloni L Riva N Meazza C . How young patients with cancer perceive the COVID-19 (Coronavirus) epidemic in milan, Italy: is there room for other fears? Pediatr Blood Cancer. (2020) 67:e28318. doi: 10.1002/pbc.28318, PMID: 32240567

[15] Colizzi M Sironi E Antonini F Ciceri ML Bovo C Zoccante L . Psychosocial and behavioral impact of COVID-19 in autism spectrum disorder: an online parent survey. Brain Sci. (2020) 10:3415. doi: 10.3390/brainsci10060341, PMID: 32503172 PMC7349059

[16] González-García H Álvarez-Kurogi L Andreu JP Cordón JT López RC Sánchez JS . Relationships among COVID-19 causal factors perceived by children, basic psychological needs and social anxiety. PeerJ. (2025) 13:e18828. doi: 10.7717/peerj.18828, PMID: 39902323 PMC11789665

[17] Polese D Costanzi F Bianchi P Gentile L Giallonardo V Buonomo C . The impact of COVID-19 on menstrual cycle’s alterations, in relation to depression and sleep disturbances: A prospective observational study in a population of medical students. BMC Women’s Health. (2024) 24:130. doi: 10.1186/s12905-024-02971-x, PMID: 38373995 PMC10877769

[18] Bronfenbrenner U . Toward an experimental ecology of human development. Am Psychol (US). (1977) 32:513−31. doi: 10.1037/0003-066X.32.7.513

[19] Qin Z Shi L Xue Y Lin H He Y Tian Z . Prevalence and risk factors associated with self-reported psychological distress among children and adolescents during the COVID-19 pandemic in China. JAMA Netw Open. (2021) 4:e2035487. doi: 10.1001/jamanetworkopen.2020.35487, PMID: 33496797 PMC7838937

[20] Gindt M Fernandez A Battista M Askenazy F . Conséquences psychiatriques de la pandémie de la Covid 19 chez l’enfant et l’adolescent. Neuropsychiatrie l’Enfance l’Adolescence. (2021) 69:115−20. doi: 10.1016/j.neurenf.2021.01.001, PMID: 33518881 PMC7837060

[21] De France K Hancock GR Stack DM Serbin LA Hollenstein T . The mental health implications of COVID-19 for adolescents: follow-up of a four-wave longitudinal study during the pandemic. Am Psychol. (2022) 77:85−99. doi: 10.1037/amp0000838, PMID: 34110880

[22] Thorisdottir IE Asgeirsdottir BB Kristjansson AL Valdimarsdottir HB Jonsdottir Tolgyes E Sigfusdottir ID . Depressive Symptoms, Mental Wellbeing, and Substance Use among Adolescents before and during the COVID-19 Pandemic in Iceland: A Longitudinal, Population-Based Study. Lancet Psychiatry. (2021) 8:663−72. doi: 10.1016/S2215-0366(21)00156-5, PMID: 34090582

[23] Ravens-Sieberer U Kaman A Erhart M Devine J Schlack R Otto C . Impact of the COVID-19 pandemic on quality of life and mental health in children and adolescents in Germany. Eur Child Adolesc Psychiatry. (2022) 31:879−89. doi: 10.1007/s00787-021-01726-5, PMID: 33492480 PMC7829493

[24] Bourion-Bédès S Tarquinio C Batt M Moret L El-Khoury L Godart N . Stress and Associated Factors among French University Students under the COVID-19 Lockdown: The Results of the PIMS-CoV 19 Study. J Affect Disord. (2021) 283:108−14. doi: 10.1016/j.jad.2021.01.041, PMID: 33540333 PMC7813474

[25] De Stefano C Laurent I Kaindje-Fondjo VC Estevez M Habran E Falissard B . Children and adolescents psychological distress scale during COVID-19 pandemic: validation of a psychometric instrument (CONFEADO study). Front Psychiatry. (2022) 13:843104. doi: 10.3389/fpsyt.2022.843104, PMID: 36003975 PMC9393589

[26] Estevez M Oppenchaim N Rezzoug D Costes JM De Stefano C Laurent I . Social determinants associated with psychological distress in children and adolescents during and after the first COVID-19-related lockdown in France: results from the CONFEADO study. BMC Public Health. (2023) 23:1374. doi: 10.1186/s12889-023-16284-5, PMID: 37464340 PMC10353123

[27] Jefferies P McGarrigle L Ungar M . The CYRM-R: A rasch-validated revision of the child and youth resilience measure. J Evidence-Based Soc Work (2019). (2018), 1−23. doi: 10.1080/23761407.2018.1548403, PMID: 30472932

[28] Peeters G . The positive-negative asymmetry: on cognitive consistency and positivity bias. Eur J Soc Psychol. (1971) 1:455−74. doi: 10.1002/ejsp.2420010405

[29] Rozin P Royzman EB . Negativity bias, negativity dominance, and contagion. Pers Soc Psychol Rev. (2001) 5:296−320. doi: 10.1207/S15327957PSPR0504_2

[30] Hamlin JK Baron. AS . Agency attribution in infancy: evidence for a negativity bias. PloS One. (2014) 9:e961125. doi: 10.1371/journal.pone.0096112, PMID: 24801144 PMC4011708

[31] Spielberger CD Edwards CD Montouri J Lushene R . State-Trait Anxiety Inventory for Children (STAI-CH) [Database record]. APA PsycTests. (1973). doi: 10.1037/t06497-000

[32] Ravens-Sieberer U Bullinger M . Assessing health-related quality of life in chronically ill children with the German KINDL: first psychometric and content analytical results. Qual Life Res. (1998) 7:399−407. doi: 10.1023/A:1008853819715, PMID: 9691720

[33] Galea S Tracy M . Participation rates in epidemiologic studies. Ann Epidemiol. (2007) 17:643−53. doi: 10.1016/j.annepidem.2007.03.013, PMID: 17553702

[34] Søgaard AJ Selmer R Bjertness E Thelle D . The Oslo Health Study: The impact of self-selection in a large, population-based survey. Int J Equity Health. (2004) 3:35. doi: 10.1186/1475-9276-3-3, PMID: 15128460 PMC428581

[35] Moreno C Wykes T Galderisi S Nordentoft M Crossley N Jones N . How mental health care should change as a consequence of the COVID-19 pandemic. Lancet Psychiatry. (2020) 7:813−24. doi: 10.1016/S2215-0366(20)30307-2, PMID: 32682460 PMC7365642

[36] Amorós-Reche V Morales A Francisco R Orgilés M Delvecchio E González-Cabrera J . Three years after the pandemic: how has the mental health of children and adolescents evolved? A longitudinal study in Italy, Spain, and Portugal. Spanish J Psychol. (2025) 28:e10. doi: 10.1017/SJP.2025.8, PMID: 40211095

[37] Mansfield KL Newby D Soneson E Calear AL Christensen H Batterham PJ . COVID-19 partial school closures and mental health problems: A cross-sectional survey of 11,000 adolescents to determine those most at risk. JCPP Adv. (2021) 1:e12021. doi: 10.1002/jcv2.12021, PMID: 34514466 PMC8420157

[38] Polsky JY Gilmour H . Food insecurity and mental health during the COVID-19 pandemic. Health Rep. (2020) 31:3−11. doi: 10.25318/82-003-x202001200001-eng, PMID: 33325672

[39] Bourion-Bédès S Rousseau H Batt M Tarquinio C Godart N El-Khoury L . The effects of living and learning conditions on the health-related quality of life of children and adolescents during the COVID-19 lockdown in the French Grand Est Region. BMC Public Health. (2022) 22:517. doi: 10.1186/s12889-022-12941-3, PMID: 35296280 PMC8926099

[40] Mbithi G Sarki A Mabrouk A Kizza I Katana E Alou T . Exploring adolescents’ Mental health in kampala, Uganda in the context of COVID-19: A mixed methods study. Front Child Adolesc Psychiatry. (2025) 4:1419043. doi: 10.3389/frcha.2025.1419043, PMID: 40065753 PMC11891163

[41] Habermann K Napp A-K Reiß F Kaman A Erhart M Ravens-Sieberer U . Supporting youths in global crises: an analysis of risk and resources factors for multiple health complaints in children and adolescents during the COVID-19 pandemic. Front Public Health. (2025) 13:1510355. doi: 10.3389/fpubh.2025.1510355, PMID: 40017543 PMC11864938

[42] Martínez-Seda GM Vélez-Pastrana MC Nicasio-Infante A . Understanding mental health impact of COVID-19 on Puerto Rican youth: influence of parental stress. Int J Environ Res Public Health. (2024) 21:125. doi: 10.3390/ijerph21121564, PMID: 39767405 PMC11675207

[43] Vallejo-Slocker L Sanz J García-Vera >MP Fresneda J Vallejo MA . Mental health, quality of life and coping strategies in vulnerable children during the COVID-19 pandemic. Psicothema. (2022) 34:249−58. doi: 10.7334/psicothema2021.467, PMID: 35485538

[44] Maunula L Dabravolskaj J Maximova K Van Lieshout R Speechley K Boyle MH . It’s very stressful for children”: elementary school-aged children’s psychological wellbeing during COVID-19 in Canada. Children. (2021) 8:1185. doi: 10.3390/children8121185, PMID: 34943381 PMC8700526

[45] Janssen LHC Kullberg M-LJ Verkuil B Wever MCM Rinck M de Vries M . Does the COVID-19 pandemic impact parents’ and adolescents’ Well-being? An EMA-study on daily affect and parenting. PloS One. (2020) 15:e0240962. doi: 10.1371/journal.pone.0240962, PMID: 33064778 PMC7567366

[46] Orgilés M Morales A Delvecchio E Mazzeschi C Espada JP Pedro MT . Coping behaviors and psychological disturbances in youth affected by the COVID-19 health crisis. Front Psychol. (2021) 12:565657. doi: 10.3389/fpsyg.2021.565657, PMID: 33828499 PMC8019796

[47] Uccella S De Grandis E De Carli F Cherubini A Furlanello F Calvo F . Impact of the COVID-19 outbreak on the behavior of families in Italy: A focus on children and adolescents. Front Public Health. (2021) 9:608358. doi: 10.3389/fpubh.2021.608358, PMID: 33614580 PMC7893111

[48] Theberath M Bauer D Chen W Kuhlmann A Roessler W Kriston L . Effects of COVID-19 pandemic on mental health of children and adolescents: A systematic review of survey studies. SAGE Open Med. (2022) 10:20503121221086712. doi: 10.1177/20503121221086712, PMID: 35371484 PMC8972920

[49] Zainel AA Daher-Nashif S Al-Maadeed AN Qotba HA Al Mujalli H Al-Kohji SM . Children and adolescents coping with home isolation and social distancing during Covid-19 in Qatar: a cross sectional study with qualitative items. BMC Psychol. (2023) 11:1505. doi: 10.1186/s40359-023-01183-6, PMID: 37149640 PMC10163849

[50] D’Amico S Marano A Geraci MA Legge E . Perceived self-efficacy and coping styles related to stressful critical life events. PloS One. (2013) 8:e675715. doi: 10.1371/journal.pone.0067571, PMID: 23874429 PMC3709911

[51] Domínguez-Álvarez B López-Romero L Isdahl-Troye A Gómez-Fraguela JA Romero E . Children coping, contextual risk and their interplay during the COVID-19 pandemic: A Spanish case. Front Psychol. (2020) 11:577763. doi: 10.3389/fpsyg.2020.577763, PMID: 33391095 PMC7772313

[52] Grills AE Ollendick TH . Multiple informant agreement and the anxiety disorders interview schedule for parents and children. J Am Acad Child Adolesc Psychiatry. (2003) 42:30−40. doi: 10.1097/00004583-200301000-00008, PMID: 12500074

[53] McGinnis EW Copeland W Shanahan L Egger. HL . Parental perception of mental health needs in young children. Child Adolesc Ment Health. (2022) 27:328−34. doi: 10.1111/camh.12515, PMID: 34653306 PMC9010484

[54] Bailey M Meiser-Stedman R Hiller R Haag K Lobo S Halligan SL . Child posttraumatic stress symptoms in an acute injury sample: patterns of associations among child report, parent report, and child heart rate parameters. J Traumatic Stress. (2023) 36:333−45. doi: 10.1002/jts.22913, PMID: 36787341 PMC10946953

[55] McDonald E Whitney S Horricks L Lipman EL Ferro MA . Parent-child agreement on the mini international neuropsychiatric interview for children and adolescents (MINI-KID). J Can Acad Child Adolesc Psychiatry =. (2021) 30:264−72., PMID: 34777509 PMC8561856

[56] Hen M Shenaar-Golan V Atia S Yatzkar U . Child-parent agreement on the SDQ: the role of child-parent attachment and parental feelings. J Clin Psychol. (2024) 80:2045−62. doi: 10.1002/jclp.23707, PMID: 38809521

[57] Massarwi AA Gross-Manos D . The association between bullying victimization and subjective well-Being among children: does the role of child religiosity matter? Int J Environ Res Public Health. (2022) 19:96445. doi: 10.3390/ijerph19159644, PMID: 35954998 PMC9367954

[58] Nordgaard J Henriksen MG Jansson L Sass LA Parnas J Møller P . Disordered selfhood in schizophrenia and the examination of anomalous self-experience: accumulated evidence and experience. Psychopathology. (2021) 54:275−81. doi: 10.1159/000517672, PMID: 34384082 PMC8686724

[59] Parnas J Møller P Kircher T Thalbitzer J Jansson L Handest P . EASE: badanie nieprawidłowego doświadczania siebie. Curr Problems Psychiatry. (2017) 18:217−41. doi: 10.1515/cpp-2017-0017

